# Comparison of artificial intelligence and logistic regression models for mortality prediction in acute respiratory distress syndrome: a systematic review and meta-analysis

**DOI:** 10.1186/s40635-024-00706-8

**Published:** 2025-02-21

**Authors:** Yang He, Ning Liu, Jie Yang, Yucai Hong, Hongying Ni, Zhongheng Zhang

**Affiliations:** 1 Department of Emergency Medicine, Sir Run Run Shaw Hospital, Zhejiang University School of Medicine, 3#, East Qingchun Road, Hangzhou, 310016 China; 2 Department of Critical Care Medicine, Affiliated Jinhua Hospital, Zhejiang University School of Medicine, No.365 Renmin East Rd, Jinhua, 321000 China; 3 Provincial Key Laboratory of Precise Diagnosis and Treatment of Abdominal Infection, School of Medicine, Sir Run Run Shaw Hospital, Zhejiang University, Zhejiang, 310016 People’s Republic of China; 4 School of Medicine, Shaoxing University, Shaoxing, China

**Keywords:** Artificial intelligence, Acute respiratory distress syndrome, Mortality, Machine learning, Neural networks

## Abstract

**Background:**

The application of artificial intelligence (AI) in predicting the mortality of acute respiratory distress syndrome (ARDS) has garnered significant attention. However, there is still a lack of evidence-based support for its specific diagnostic performance. Thus, this systematic review and meta-analysis was conducted to evaluate the effectiveness of AI algorithms in predicting ARDS mortality.

**Method:**

We conducted a comprehensive electronic search across *Web of Science, Embase, PubMed, Scopus*, and *EBSCO* databases up to April 28, 2024. The QUADAS-2 tool was used to assess the risk of bias in the included articles. A bivariate mixed-effects model was applied for the meta-analysis. Sensitivity analysis, meta-regression analysis, and tests for heterogeneity were also performed.

**Results:**

Eight studies were included in the analysis. The sensitivity, specificity, and summarized receiver operating characteristic (SROC) of the AI-based model in the validation set were 0.89 (95% CI 0.79–0.95), 0.72 (95% CI 0.65–0.78), and 0.84 (95% CI 0.80–0.87), respectively. For the logistic regression (LR) model, the sensitivity, specificity, and SROC were 0.78 (95% CI 0.74–0.82), 0.68 (95% CI 0.60–0.76), and 0.81 (95% CI 0.77–0.84). The AI model demonstrated superior predictive accuracy compared to the LR model. Notably, the predictive model performed better in patients with moderate to severe ARDS (SAUC: 0.84 [95% CI 0.80–0.87] vs. 0.81 [95% CI 0.77–0.84]).

**Conclusion:**

The AI algorithms showed superior performance in predicting the mortality of ARDS patients and demonstrated strong potential for clinical application. Additionally, we found that for ARDS, a highly heterogeneous condition, the accuracy of the model is influenced by the severity of the disease.

## Introduction

Acute respiratory distress syndrome (ARDS) is a severe condition characterized by profound respiratory failure and a high mortality rate. In clinical practice, ARDS rarely occurs in isolation; rather, it often develops as a consequence of underlying conditions such as sepsis, trauma, pancreatitis, and other related diseases. The Berlin criteria define ARDS based on acute lung injury, bilateral chest infiltrates, and hypoxemia that cannot be fully attributed to other causes [1, 2]. For clinicians, reliable mortality prediction in ARDS patients is crucial [3–5]. Despite the existence of predictive models that incorporate multiple variables influencing prognosis, accurately forecasting mortality in ARDS patients remains a significant challenge [5–7].

Artificial intelligence (AI) is an emerging technology that has demonstrated strong performance across various industries. One of AI's key capabilities is its ability to build predictive models. Numerous studies have investigated the AI-based models for predicting ARDS mortality [8–15]. However, several questions remain unresolved: (1) the diagnostic performance of these models varies considerably across studies, and it’s uncertain whether an AI-based model can consistently outperform traditional linear models in mortality prediction; (2) it is unclear whether the characteristics of the data (e.g., data size or severity of the disease) used affect model performance; and (3) there might be biases in the mortality predictions generated by these AI models.

To address these gaps, this study systematically reviewed the existing literature to evaluate the performance of AI algorithms in predicting ARDS mortality, with the hope that our findings will contribute to the advancement and application of AI in this field.

## Methods

### Selection criteria

This systematic review and meta-analysis adhered to PRISMA-DTA standards, and the study protocol was registered [INPLASY 202450005, http://www.inplasy.com].

**Inclusion criteria:**

- Patients aged over 18 years;
- Patients diagnosed with ARDS according to the Berlin definition;
- The prediction models were developed using AI or LR algorithms, and two or more variables were included;
- Collection of modeling variables within 48 h after ARDS diagnosis;
- Models must be validated either internally or externally.

In AI research, conducting independent external validation can be challenging due to limited resources; therefore, methods like K-fold cross-validation or the leave-one-out approach are commonly used. While these methods are valuable, we must also consider the risk of overfitting from an evidence-based perspective. Hence, studies lacking even internal validation were excluded.

**Exclusion criteria:**

- Use of specialized molecular markers that are not readily available during the modeling process;
- Missing necessary data;
- Other types of articles, such as meta-analysis, review, guideline, and expert comments.

### Search strategy

We searched Web of *Science, Embase, PubMed, Scopus* and *EBSCO* in collaboration with a specialist librarian from inception to April 28, 2024. The search strategy was: *((ARDS) OR (acute lung injury) OR (acute respiratory distress syndrome)) AND (mortality) AND (prediction) AND ((AUC) OR (sensitivity) OR (specificity))*.

### Study screening and data extraction

Three authors (Yang He, Ning Liu, and Jie Yang) independently conducted the initial screening using the EndNote20 reference management software. After removing duplicates and reviewing titles and abstracts, studies that met the eligibility criteria underwent a full-text assessment by at least two of the authors (Yang He, Ning Liu, or Jie Yang). Any discrepancies were resolved by a fourth author (Zhongheng Zhang). Before finalizing the list of studies for meta-analysis, all studies were examined to check for duplicate cohorts.

We included studies that satisfied the predefined eligibility criteria based on the full-text review. A standardized form was used for data extraction, capturing essential details such as the title, author, publication date, nationality, study type, algorithm used, modeling data type (clinical data, imaging data, or both), cohort size, validation type (internal or external validation), ARDS severity, cause of ARDS, and counts of true positives (TP), false negatives (FN), false positives (FP), and true negatives (TN). Some studies reported multiple machine learning algorithms. In such cases, we included the data from the algorithm that showed the best performance.

### Quality assessment

We independently evaluated all included studies using the modified quality assessment of diagnostic accuracy studies (QUADAS-2) tool [16]. This assessment focused on applicability to the review question and risk of bias. The evaluation was conducted by at least two authors (Yang He, Ning Liu, Jie Yang, or Zhongheng Zhang).

### Statistical analysis

First, we performed subgroup the analyses and regression analyses on the included models to identify factors influencing their sensitivity and specificity.

Second, we conducted a meta-analysis of diagnostic tests for the subgroups formed by different factors. We utilized a bivariate mixed-effects model for the meta-analysis. This model accounts for both fixed and random effects, effectively managing heterogeneity across studies and addressing the correlation between sensitivity (SEN) and specificity (SPE), resulting in more robust and reliable results [17]. We compared model accuracy across the subgroups. The bivariate mixed-effects model incorporated SEN and SPE, along with the negative likelihood ratio (NLR), positive likelihood ratio (PLR), diagnostic odds ratio (DOR), and 95% confidence intervals (95% CI). We also generated a summarized receiver operating characteristic (SROC) curve and calculated the area under the curve (AUC). Additionally, Deek’s funnel plot was used to assess publication bias.

All the data analyses were performed using Stata (version: 18.0) and a two-tailed *p* < 0.05 implied statistical significance.

## Results

### Search outcome and study characteristics

A total of 2536 results were identified in the initial search.

**Fig. 1:**
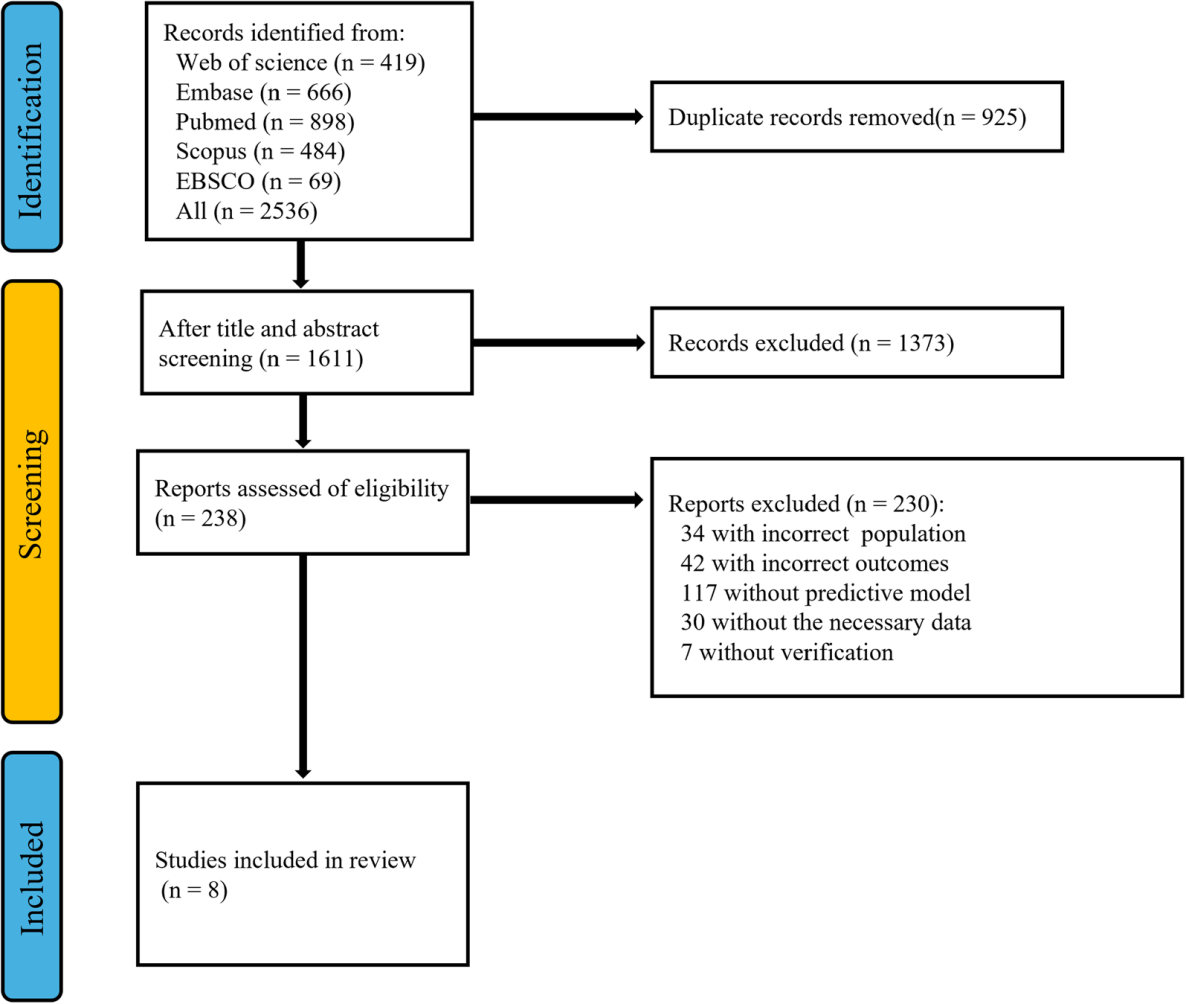
Flow diagram of the study selection process

925 studies were excluded as duplicates and a further 1373 studies were excluded during screening of titles and abstracts. The remaining 238 texts were reviewed in full, and a further 230 studies were excluded (Fig. 1). In total, datasets from 8 studies [8–14, 18], involving 5664 patients and published between 2017 and 2024, were included in this meta-analysis. All included studies were retrospective, and three of them utilized data from the MIMIC III database [11, 12, 18]. Among these, two studies employed unique database queries for modeling [11, 12], one study utilized different external data for validation purposes [18]. In one study cohort, sepsis was identified as the cause of ARDS [8], while trauma was implicated in another study cohort [13]. The remaining studies did not specify the etiology of ARDS. Four studies compared the predictive power of AI and LR models [10, 13, 14, 18]. Image data (e.g., CT scans or X-rays) were utilized in the model development of two studies [8, 9]. Except for one study, which used 90-day mortality as the endpoint [14], the other studies focused on short-term all-cause mortality in ARDS patients.

The characteristics of each study are described in Table 1.

**Table 1:** Characteristics of the studies included in the meta-analysis

Author	Country	Algorithm	Modeling data	Modeling data size	Verification cohort size/verification type	Severity of ARDS	Cause of ARDS	Mortality calculation time	TP/FN/FP/TN
Cysneiros et al. 2024	Portugal	AI	Imaging and clinical data	85	23/Internal verification	Moderate and severe	Sepsis	In-hospital	9/1/4/9
Wang et al. 2023	China	AI	Imaging and clinical data	256	110/Internal verification	All	Mixed	28d	25/2/34/49
Villar et al. 2023	Spain	AI and LR	Clinical data	1000	303/External verification	Moderate and severe	Mixed	ICU	99/14/37/153 95/17/31/160
Zhang et al. 2023	China	AI	Clinical data	2807	1203/internal verification	Moderate and severe	Mixed	In-hospital	860/17/130/196
Wang et al. 2022	China	LR	Clinical data	1230	584/internal verification 168/external verification	All	Mixed	28d	217/53/152/232 59/19/32/58
Tang et al. 2022	China	AI and LR	Clinical data	532	228/internal verification	All	Trauma	In-hospital	29/14/49/139 33/10/75/110
Hu et al. 2022	China	AI and LR	Clinical data	477	143/internal verification	All	Mixed	In-hospital	54/11/11/64 44/21/21/56
Zhang et al. 2019	China	AI and LR	Clinical data	745	272/ external verification	All	Mixed	90d	114/29/35/94 109/34/41/88

### Methodological quality

**Fig. 2:**
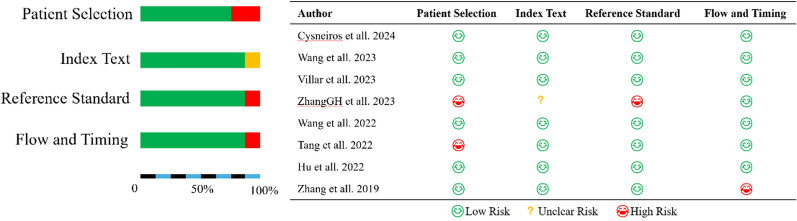
Methodological assessment by QUADAS-2

Regarding the QUADAS-2 risk-of-bias assessment (Fig. 2), we found 3 studies with a high risk of bias [11, 12, 14], and the rest were of high quality.

### Subgroup analyses and meta-regression analysis

We performed the subgroup analyses in four aspects, including algorithm (AI or LR), modeling cohort size (*n* < 1000 or *n* > 1000), verification type (internal verification or external verification) and severity of ARDS. This was done to better understand how these factors influenced the performance of the predictive models for ARDS mortality (Table 2). The algorithm type, cohort size, and ARDS severity significantly affected the specificity of the model (*P* < 0.05), whereas the sensitivity of the model was primarily influenced by the validation type and disease severity (*P* < 0.05).

**Table 2:** Subgroup analyses stratified by various cohort characteristics

Parameter	Category	N-cohorts	Sensitivity	p1	Specificity	p2
Algorithm	AI	7	0.89 [0.83–0.95]	0.42	0.72 [0.66–0.79]	0.03*
	LR	6	0.78 [0.67–0.88]		0.69 [0.61–0.76]	
Cohort size	< 1000	8	0.79 [0.70–0.89]	0.29	0.70 [0.63–0.77]	0.02*
	> 1000	5	0.81 [0.71–0.92]		0.75 [0.68–0.82]	
Verification type	Internal verification	8	0.85 [0.78–0.93]	0.03*	0.67 [0.61–0.74]	0.05
	External verification	5	0.81 [0.71–0.92]		0.75 [0.68–0.82]	
Severity of ARDS	All	9	0.78 [0.71–0.85]	0.00*	0.68 [0.62–0.74]	0.00*
	Only moderate and severe	4	0.93 [0.89–0.97]		0.76 [0.68–0.83]	

Meta-regression analysis was also conducted by adjusting for different cohort characteristics (Table 3). We found that the severity of ARDS within the cohort significantly affected the model’s predictive accuracy (*P* < 0.05). The algorithm type (AI versus LR) showed a potential influence on the model’s accuracy (*P* < 0.1).

**Table 3:** Meta-regression analysis to adjust for different cohort characteristics

Parameter	Category	LRT Chi2	*P* value	I2	I2lo	I2hi
Algorithm	AI	4.79	0.09	58	6	100
	LR					
Cohort size	< 1000	2.34	0.31	14	0	100
	> 1000					
Verification type	Internal verification	2.31	0.32	13	0	100
	External verification					
Severity of ARDS	All	15.81	0.00*	87	74	100
	Only moderate and severe					

### Comparison of the AI and LR prediction model

A total of 7 studies reported on AI models for predicting ARDS prognosis, while 6 studies reported on LR models for the same purpose. The pooled sensitivity and specificity of the AI models were 0.89 (95%CI 0.79–0.95) and 0.72 (95%CI 0.65–0.78). The PLR, NLR, and DOR were 3.22 (95% CI 2.64–3.93), 0.15 (95% CI 0.08–0.29), and 21.57 (95% CI 11.21–41.49), respectively.

**Fig. 3:**
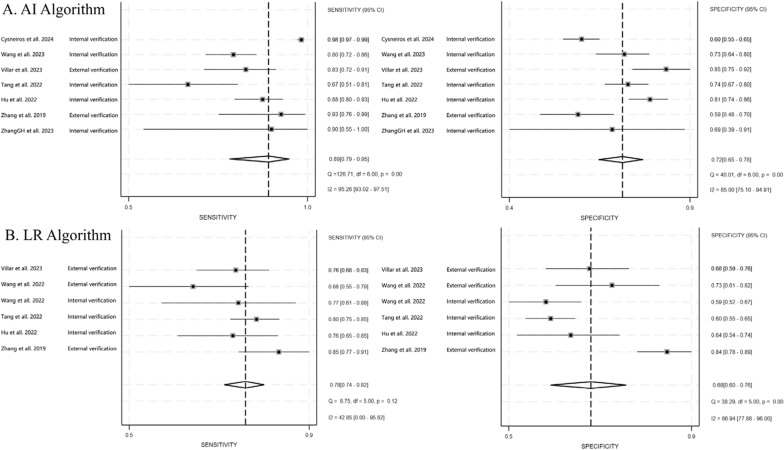
Paired forest plots for the pooled sensitivity and specificity of each algorithm for the prediction of ARDS mortality. **A** AI model and **B** LR model

For LR models, the pooled sensitivity and specificity were 0.78 (95%CI 0.74–0.82) and 0.68 (95% CI 0.60–0.76). The PLR, NLR, and DOR were 2.48 (95% CI 1.91–3.22), 0.32 (95% CI 0.25–0.40), and 7.79 (95% CI 4.84–12.53), respectively. Overall, the AI models demonstrated higher sensitivity, specificity, and DOR compared to the LR models (Fig. 3).

**Fig. 4:**
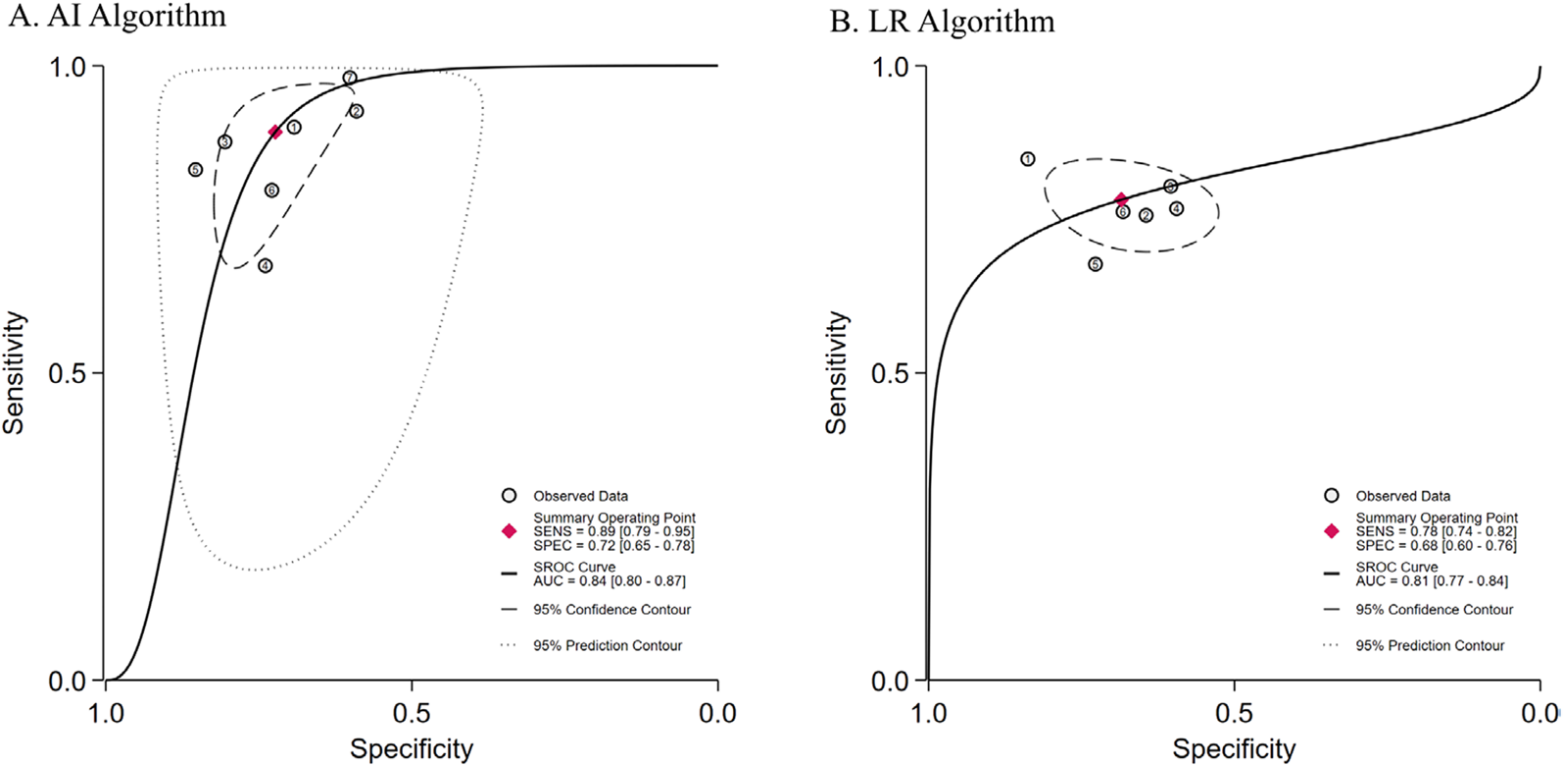
The SROC for prediction of ARDS mortality with AI and LR

The SROC for predicting ARDS mortality using AI and LR models are presented in Fig. 4. The SAUC of the AI model was higher than that of the LR model (SAUC: 0.84 [95% CI 0.80–0.87] vs. 0.81 [95% CI 0.77–0.84]), indicating a better predictive performance. No significant publication bias was detected (*P* > 0.05) (Figure S1).

### Influence of the severity of ARDS on the prediction model

**Fig. 5:**
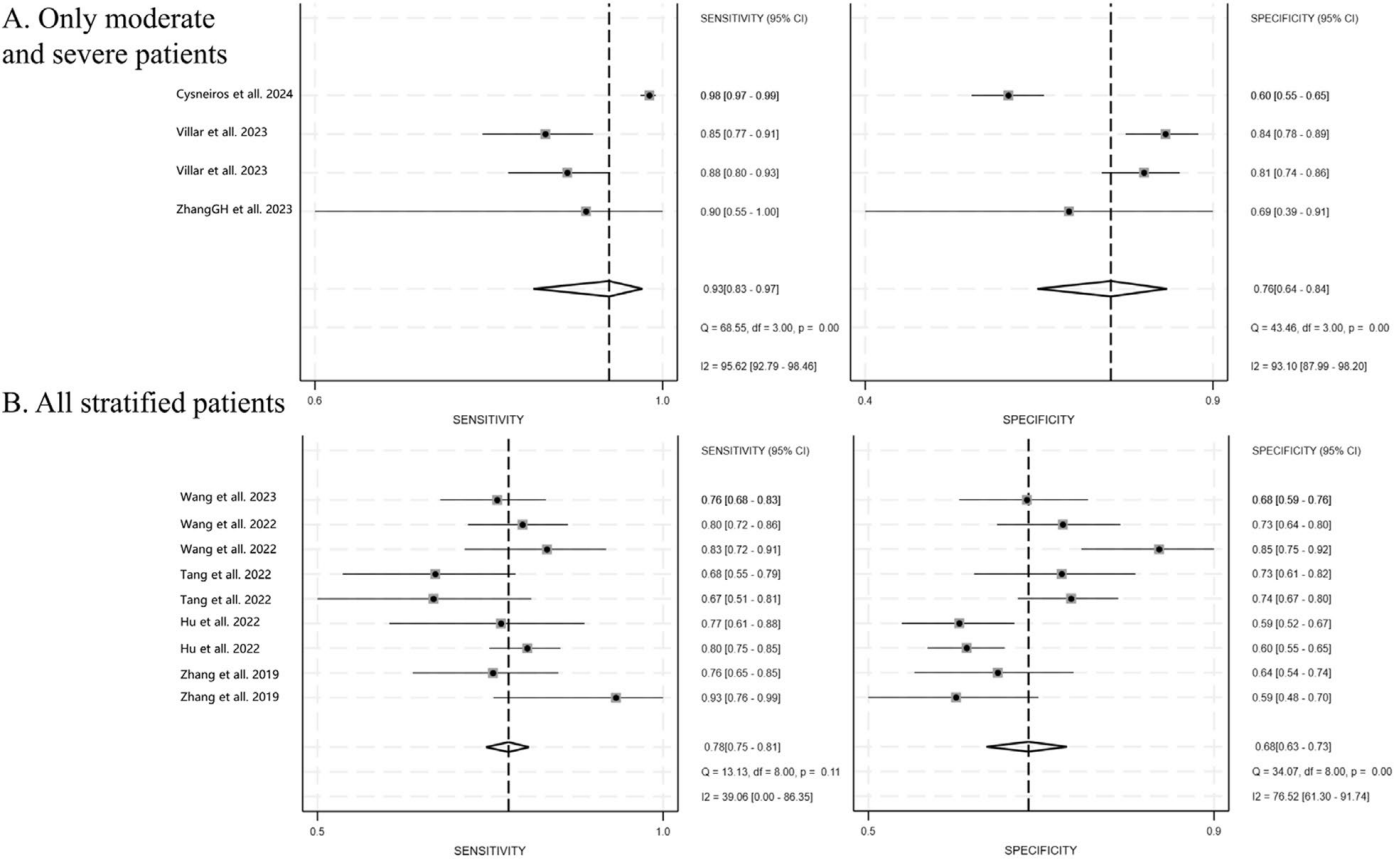
Paired forest plots for the pooled sensitivity and specificity of models for the prediction of mortality of ARDS with different severity. **A** Moderate and severe ARDS cohort and **B** all stratified patients cohort

We evaluated the impact of ARDS severity on the predictive model (Fig. 5). In studies focusing on moderate and severe ARDS cases, the pooled sensitivity and specificity of the model were 0.93 (95% CI 0.83–0.97) and 0.76 (95% CI 0.64–0.84), respectively. The PLR, NLR, and DOR were 3.80 (95% CI 2.70–5.35), 0.09 (95% CI 0.04–0.21), and 40.66 (95% CI 23.45–70.50), respectively.

For all stratified patients, the pooled sensitivity and specificity were 0.78 (95% CI 0.75–0.81) and 0.68 (95% CI 0.63–0.73), respectively. The PLR, NLR, and DOR were 2.46 (95% CI 2.10–2.89), 0.32 (95% CI 0.28–0.37), and 7.59 (95% CI 5.87–9.81), respectively.

**Fig. 6:**
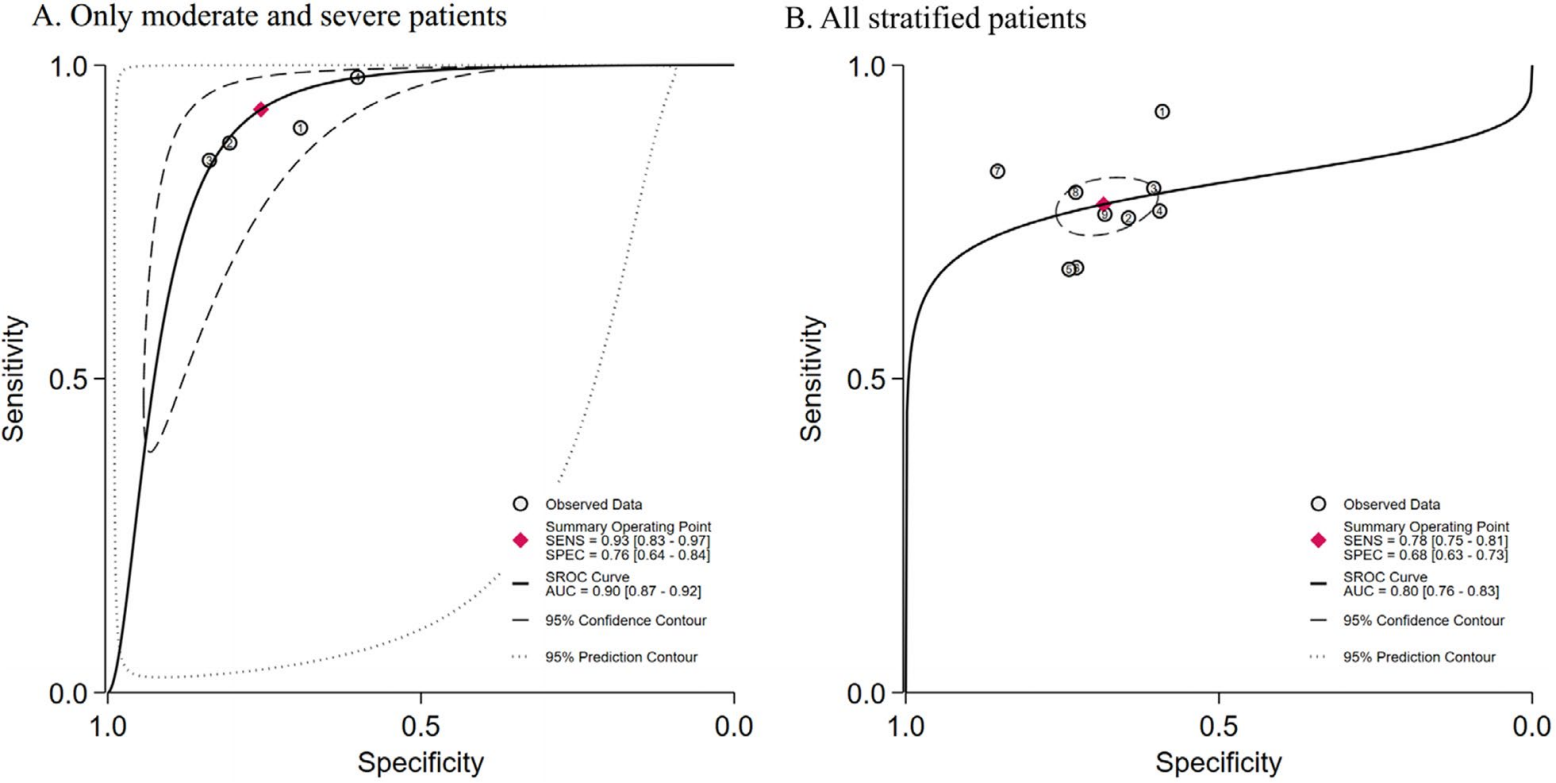
The SROC for prediction model of ARDS mortality based on different severity

Notably, these differences were also reflected in the SROC curve. The SAUC of the model based on the moderate and severe ARDS group is larger than that of the model based on all stratified patients (AUC 0.90 [0.87–0.92] vs. 0.80 [0.76–0.83]), indicating better predictive performance (Fig. 6). No evident publication bias was noticed (*P* > 0.05) (Figure S2).

## Discussion

In this study, we performed a systematic review of articles focused on clinical data-based models for predicting ARDS mortality. We evaluated the predictive value of AI models and compared their performance with traditional LR models. Furthermore, we examined the accuracy of these models across cohorts with varying levels of ARDS severity.

Our research suggests that AI algorithms exhibit higher accuracy in constructing predictive models compared to traditional LR algorithms. This increased accuracy is largely attributed to the superior computational power and precise data processing capabilities of AI algorithms. In numerous studies, it remains uncertain whether AI algorithms consistently outperform LR algorithms. Lynam et al. compared the performance of LR algorithms with six optimized AI algorithms in distinguishing between type 1 and type 2 diabetes patients. Their results indicated that LR algorithms performed comparably to other AI algorithms across both internal and external validation datasets and even outperformed them in certain instances [19]. Similarly, Shariati et al. examined the performance of artificial neural networks and multivariable LR models in predicting outcomes for patients with head trauma. Their findings revealed that both models achieved similar predictive performance, though the LR model offered greater interpretability [20]. Our previous research on the predictive ability of AI algorithms for ARDS mortality indicated that AI algorithms performed comparably to LR algorithms [14]. Through this meta-analysis, we sought to obtain more definitive and compelling evidence. The results reveal that the predictive capability of AI algorithms indeed surpasses that of LR algorithms.

However, it is worth noting that the performance of AI varied across the studies included in our analysis (Fig. 3A), potentially due to differences in hyperparameter tuning during the modeling process. Researchers from different disciplines may employ distinct approaches to hyperparameter optimization. Someone with a background in engineering might focus more on describing algorithmic details, while physicians might focus more on the relevant aspects for clinical practice. Such discrepancies can lead to issues like overfitting or underfitting, which may affect the practical utility and adoption of the models [21, 22].

In our study, the accuracy of the diagnostic model varied significantly across different ARDS severity cohorts. This discrepancy may be attributed to the high heterogeneity observed in ARDS cases. Previous research has identified two distinct subtypes of ARDS: hypoinflammatory and hyperinflammatory. These phenotypes exhibit differential outcomes and responses to treatment [23–27]. Additionally, sepsis, another common critical illness, also displays considerable heterogeneity. Our research team employed an unsupervised learning algorithm to explore sepsis subtypes based on clinical features. We identified four sepsis subtypes: basal type, respiratory failure type, multiple organ failure type, and neurological failure type. Further investigations revealed that patients within these subtypes had varying prognoses and responded differently to fluid therapy. For instance, patients in multiple organ failure type may benefit from appropriately increased daily fluid intake [28]. Recognizing these subtypes is crucial for developing accurate prediction models for ARDS mortality.

During our search process, we found that numerous studies have explored the use of molecular markers for predicting ARDS mortality. For example, one study reported that combining miR-92a and miR-146a with pulmonary ultrasound scores achieved an AUC of 0.918, a SEN of 0.94, and a SPE of 0.852 [29]. Another study evaluated autotaxin in combination with pulmonary ultrasound scores, yielding an AUC of 0.904, a SEN of 0.938, and a SPE of 0.806 [30]. Furthermore, other molecular markers such as plasma matrix metalloproteinase-3, soluble vascular endothelial growth factor receptor (sFlt-1), angiopoietin-2 (Ang-2), von Willebrand factor (vWF), and Clara cell secretory protein 16 (CC16) have also been linked to ARDS mortality prediction [31–34]. Integrating molecular biomarkers into predictive models could significantly improve their accuracy. Additionally, exploring multi-omics and multi-modal data is a promising strategy for further enhancing predictive performance [35, 36].

This study has several limitations. Firstly, there is significant heterogeneity across the included studies. However, this should not be viewed as a reason to discount the findings, as such heterogeneity is an inherent aspect of the AI modeling process. Due to the limited number of studies, we were unable to perform subgroup analyses for different AI algorithms (e.g., CNN, SVM). Moreover, the data used in these studies come from various medical institutions with differing levels of healthcare quality, which may result in variations in patient outcomes. Unfortunately, we did not have sufficient data to stratify and analyze the ARDS prognostic prediction models based on the level of medical institutions. These real-world variations can affect the accuracy of the models. Secondly, all the included studies were retrospective in nature. Retrospective studies often face challenges such as incomplete data collection, lower data quality, and inherent biases, which can compromise the reliability and generalizability of the findings [22, 37].

## Conclusion

This meta-analysis demonstrates that AI-based models exhibit excellent performance in predicting the prognosis of ARDS, with both sensitivity and specificity surpassing those of traditional LR models. AI-based models hold significant potential for clinical application and could serve as a valuable adjunct tool to assist clinicians in making earlier interventions.

## Data Availability

The data that support the findings of this study are available from the corresponding author upon reasonable request.

## Abbreviations

AI: Artificial intelligence
TP: True positive
FP: False positive
TN: True negative
FN: False negative
NLR: Negative likelihood ratio
PLR: Positive likelihood ratio
DOR: Diagnostic odds ratio
95% CI: 95% Confidence intervals
SROC: Summarized receiver operator characteristic
